# 5-aminoimidazole-4-carboxamide-1-beta-4-ribofuranoside (AICAR) attenuates the expression of LPS- and Aβ peptide-induced inflammatory mediators in astroglia

**DOI:** 10.1186/1742-2094-2-21

**Published:** 2005-09-20

**Authors:** Kamesh R Ayasolla, Shailendra Giri, Avtar K Singh, Inderjit Singh

**Affiliations:** 1 Department of Pediatrics, Medical University of South Carolina, Charleston, South Carolina, 29425, USA; 2 Department of Pathology, Medical University of South Carolina, Charleston, South Carolina, 29425, USA; 3 Department of Obstetrics & Gynaecology, Medical University of South Carolina, Charleston, South Carolina, 29425, USA; 4 Department of Pathology, Ralph H. Johnson VA Medical Center, Charleston, South Carolina 29425, USA

## Abstract

**Background:**

Alzheimer's disease (AD) pathology shows characteristic 'plaques' rich in amyloid beta (Aβ) peptide deposits. Inflammatory process-related proteins such as pro-inflammatory cytokines have been detected in AD brain suggesting that an inflammatory immune reaction also plays a role in the pathogenesis of AD. Glial cells in culture respond to LPS and Aβ stimuli by upregulating the expression of cytokines TNF-α, IL-1β, and IL-6, and also the expression of proinflammatory genes iNOS and COX-2. We have earlier reported that LPS/Aβ stimulation-induced ceramide and ROS generation leads to iNOS expression and nitric oxide production in glial cells. The present study was undertaken to investigate the neuroprotective function of AICAR (a potent activator of AMP-activated protein kinase) in blocking the pro-oxidant/proinflammatory responses induced in primary glial cultures treated with LPS and Aβ peptide.

**Methods:**

To test the anti-inflammatory/anti-oxidant functions of AICAR, we tested its inhibitory potential in blocking the expression of pro-inflammatory cytokines and iNOS, expression of COX-2, generation of ROS, and associated signaling following treatment of glial cells with LPS and Aβ peptide. We also investigated the neuroprotective effects of AICAR against the effects of cytokines and inflammatory mediators (released by the glia), in blocking neurite outgrowth inhibition, and in nerve growth factor-(NGF) induced neurite extension by PC-12 cells.

**Results:**

AICAR blocked LPS/Aβ-induced inflammatory processes by blocking the expression of proinflammatory cytokine, iNOS, COX-2 and MnSOD genes, and by inhibition of ROS generation and depletion of glutathione in astroglial cells. AICAR also inhibited down-stream signaling leading to the regulation of transcriptional factors such as NFκB and C/EBP which are critical for the expression of iNOS, COX-2, MnSOD and cytokines (TNF-α/IL-1β and IL-6). AICAR promoted NGF-induced neurite growth and reduced neurite outgrowth inhibition in PC-12 cells treated with astroglial conditioned medium.

**Conclusion:**

The observed anti-inflammatory/anti-oxidant and neuroprotective functions of AICAR suggest it as a viable candidate for use in treatment of Alzheimer's disease.

## Background

Alzheimer's disease (AD) is a neurological disorder and the brain pathology is characterized by the presence of senile plaques rich in insoluble aggregates of beta amyloid (1–40) and (1–42) peptides, degradation products of the larger amyloid precursor protein (APP) [1,2]. All major pro-inflammatory cytokines with the exception of IFN-γ (TNF-α, IL-1 and IL-6) have been detected in AD brain suggesting that an inflammatory immune reaction also plays a role in the pathogenesis of AD [3,4]. The deposited Aβ peptides have also been implicated in oxidative stress-induced responses, via NADPH oxidase activation and superoxide anion generation [5].

The astroglial population has a major role in neuroinflammatory disease processes, and has been implicated in various neurological disorders including AD [6]. Though we still do not know what endogenous ligands may trigger an inflammatory response in AD, several studies have reported that LPS/Aβ treatment of glia serves as a good cell culture model for mimicking the inflammatory conditions in AD [7-10]. In vitro treatment of glial cells with LPS/Aβ peptides induces cytokines (TNF-α, IL-1β), and also leads to the release of NO by induction of iNOS, as a function of innate immune response (for a detailed review see [6,11]). COX-2, an enzyme in the PLA-2 cascade, involved in the arachidonic acid metabolic pathways for the synthesis of prostaglandins, is yet another enzyme that is expressed along with other inflammatory mediators in these glial cells [6]. Its expression has been observed to be coincident with the onset of expression of apoptotic neuronal cell death markers, due to excitotoxic neurotoxicity. The expression of iNOS leading to production of nitric oxide and, as a result, generation of peroxynitrite (a reaction product of the superoxide anion and nitric oxide) under oxidative stress conditions has also been implicated in the extensive neuronal damage of several neurological disorders including AD [12,13]. Therefore the mechanisms of pro-inflammatory cytokine-mediated oxidative stress (or *vice versa*) may be the potential target(s) for AD therapeutics.

AMP-activated protein kinase [14] (AMPK) is a member of the family of serine/threonine kinases and is activated by cellular increases in AMP concentrations under conditions of nutritional/metabolic stress [15].

This is thus often referred to as the fuel gauge of the cell, since it protects the cell against ATP depletion and boosts the energy generation pathways [16,17]. AMPK is activated by AMP-dependent phosphorylation by an upstream kinase, i.e. AMPK kinase (AMPKK; recently recognized as LKB1 [16,17]. AICAR is also reported to activate AMPK in the cell following its conversion to ZMP (a non-degradable AMP analog) and thus mimics the activity of AMP for activation of AMPK [18]. Recently, we reported anti-inflammatory properties of AICAR through activation of AMPK [19] in glial cells. AICAR was found to inhibit expression of pro-inflammatory cytokines and of iNOS in glial cells and in macrophages in cell culture as well as in rats treated with a sublethal dose of LPS [19] by attenuating NFκB and C/EBP pathways.

Aβ peptides are known to alter cellular redox, thereby triggering down stream kinase cascades leading to inflammation [12,20]. Hence this study was designed to evaluate the anti-oxidant/anti-inflammatory functions of AICAR in blocking LPS/Aβ-mediated down-stream signaling cascades leading to transcription factor activation and inflammatory cytokine release and iNOS and COX-2 expression. This study describes AICAR-mediated activation of AMPK and downregulation of LPS/Aβ-induced expression of inflammatory mediators in astrocyte-enriched glial cell cultures, possibly via reduction/regulation of cellular redox.

## Methods

### Reagents

DMEM and fetal bovine serum were obtained from Life Technologies Inc., Gaithersburg MD, USA, and LPS (Escherichia coli) from Calbiochem. Antibodies against iNOS and MnSOD were obtained from Transduction Labs, and antibody to COX-2 was from Cayman chemicals, Ann Arbor, MI. β-Actin and β-amyloid peptide (25–35) fragment as well as the reverse peptide (35–25), β-αmyloid peptides (1–40) and (1–42) were from Sigma. Antibodies for p65; p50; IB kinase (KKK); CCAAT/enhancer-binding proteins (C/EBP)-α, -β, and -δ; and oligonucleotides for NF-κB and C/EBP were from Santa Cruz. Recombinant tumor necrosis factor (TNF-α) and interleukin (IL)-1; and ELISA kits for TNF-α, IL-1, IL-6, and IFN-γ were from R & D Systems. Trizol and transfection reagents (Lipofectamine-2000, Lipofectamine-Plus, and Oligofectamine) were from Invitrogen. Chloramphenicol acetyltransferase ELISA, -galactosidase (-gal), 3-(4,5-dimethylthiazol-2-yl)-2,5-diphenyl tetrazolium bromide (MTT), and lactic dehydrogenase (LDH) kits were obtained from Roche. The enhanced chemiluminescence-detecting reagents were purchased from Amersham Biosciences. The luciferase assay system was from Promega. Antibodies against phosphospecific as well as nonphospho-p42/44, and -AMPK were from Cell Signaling Technology. NF-κB-luciferase was provided by Dr. Hanfang Zhang (Medical College of Georgia, Augusta, GA).

### Cell culture and treatment of rat primary glial cultures and astrocytes

Astroglial cells were isolated from rat cerebral tissue as described by McCarthy and DeVellis [21]. Astrocytes were isolated and maintained as described earlier [12]. Cells were maintained in DMEM containing 10% fetal bovine serum. Glial cells were stimulated with either LPS (125 μg/ml), cytokines, or with sphingomyelinase (SMase) with or without β-amyloid peptide in serum-free DMEM and were harvested after 18 h unless stated otherwise. AICAR (1 mM), NAC (15 mM), Vitamin E (20 μM), or other substances were added 4 hr prior to stimulation with LPS/cytokines and were again added at the time of addition of stress stimuli.

### Preparation of aged Aβ (1–40), (1–42) and (25–35) and induction of cells with β-amyloid peptide

The Aβ peptides (25–35), (1–40), (1–42) and the reverse peptide Aβ (40–1) were all purchased from Sigma. They were solubilized in phosphate-buffered saline (PBS) at a concentration of 1 mM, incubated in a capped vial at 37°C for 4 days [22], and stored frozen at -20°C until use. They were used at a final concentration of 7.5 μM or in higher amounts, as indicated.

### Assay for NO synthesis

Synthesis of NO was determined by assaying culture supernatants for nitrite, a stable reaction product of NO with molecular oxygen [19]. Briefly, 400 μl of culture supernatant was allowed to react with 200 μl of Griess reagent and incubated at room temperature for 15 min. The optical density of the assay samples was measured spectrophotometrically at 570 nm. Fresh culture media served as the blank in all experiments. Nitrite concentrations were calculated from a standard curve derived from the reaction of NaNO_2 _in the assay.

### Fluorescence measurements for superoxide production using hydroethidine

Hydroethidine (HE) or dihdroethidium (DHE), a redox sensitive probe, have been widely used to detect intracellular superoxide anion. The oxidation of HE in a superoxide generating system was performed by spectrofluorimetry, essentially according to the method described by Zhao, et al [23] with slight modifications. Briefly, following treatment of cells with LPS, with or without Aβ ± AICAR (1 mM), for 6 h, the cultures were rinsed in PBS and the medium was replaced with fresh medium containing 50 μM HE (stock solution 5 mM in dimethyl sulfoxide) in DMEM/high glucose-containing medium. Following incubation for 60 min at 37°C, cells were rinsed twice in phosphate-buffered saline (PBS) to remove any unbound dye and then lysed in buffer containing 0.1 N NaOH in 50% MetOH and vortexed for 20 min on a shaker. Generation of ROS was measured by a fluorescence plate reader, at an excitation wavelength of 510 nm, and emission at 595 nm (gain 10). The blank values consisted of wells containing no cells but loaded with HE and identically processed. Equal volumes of PBS or NaOH-MetOH were added for cell lysis, before fluorescence measurement.

### Immunoblot analysis

These were performed essentially as described earlier [12,19]. Briefly, glial cells (2 × 10^6^/ml), after incubation in the presence or absence of different stimuli, cell lysates was prepared in 0.5 ml of buffer containing 20 mM HEPES, pH 7.4, 2 mM EDTA, 250 mM NaCl, 0.1% Nonidet, P-40, 0.1% Triton-X (100), 2 μg/ml leupeptin, 2 μg/ml aprotinin, 1 mM phenylmethylsulfonyl fluoride, 0.5 μg/ml benzamidine, and 1 mM dithiothreitol. The lysate was briefly centrifuged at 500 rpm for 10 min, and the supernatant was collected. Cell extract protein (50 μg) was then resolved on 4–10% SDS-PAGE, electrotransferred onto a nitrocellulose membrane, blotted with indicated antibodies, and then detected by chemiluminescence (ECL; Amersham Pharmacia Biotech).

### Preparation of nuclear extracts and electrophoretic mobility shift assay (EMSA)

Nuclear extracts from treated or untreated cells (1 × 10^7^) were prepared using the method of Dignam et al, [24] with slight modification. Cells were harvested, washed twice with ice-cold PBS, and lysed in 400 μl of buffer A (10 mM HEPES, pH 7.9; 10 mM KCl; 2 mM MgCl2; 0.5 mM dithiothreitol; 1 mM phenylmethylsulfonyl fluoride; 5 μg/ml aprotinin; 5 μg/ml pepstatin A; and 5 μg/ml leupeptin) containing 0.1% Nonidet P-40 for 15 min on ice, vortexed vigorously for 15 s, and centrifuged at 14,000 rpm for 30 s. The pelleted nuclei were resuspended in 40 μl of buffer B (20 mM HEPES, pH 7.9; 25% (v/v) glycerol; 0.42 M NaCl; 1.5 mM MgCl_2_; 0.2 mM EDTA; 0.5 mM dithiothreitol; 1 mM phenylmethylsulfonyl fluoride; 5 μg/ml aprotinin; 5 μg/ml pepstatin A; and 5 μg/ml leupeptin). After 30 min on ice, the lysates were centrifuged at 14,000 rpm for 10 min. Supernatants containing the nuclear proteins were diluted with 20 μl of modified buffer C (20 mM HEPES, pH 7.9; 20% (v/v) glycerol; 0.05 M KCl; 0.2 mM EDTA; 0.5 mM dithiothreitol; and 0.5 mM phenylmethylsulfonyl fluoride) and stored at -70°C until further use. Nuclear extracts were used for the electrophoretic mobility shift assay using the NFκB DNA-binding protein detection system kit (Life Technologies, Inc.) according to the manufacturer's protocol. Briefly, the protein-binding DNA sequences (previously labeled with ^32^P) of C/EBP, NFκB, AP-1 and CREB were incubated with nuclear extracts prepared after various treatments of glial cells. The DNA-protein binding reactions were performed at room temperature for 20 min in 10 mM Trizma base pH 7.9, 50 mM NaCl, 5 mM MgCl_2_, 1 mM EDTA, and 1 mM dithiothreitol plus 1 μg of poly (dI-dC), 5% (v/v) glycerol, and ~0.3 pmol of ^32^P labeled either C/EBP, NFκB, AP-1 or CREB (all from Santa Cruz Biotechnology). Protein DNA complexes were resolved from protein-free DNA in 5% polyacrylamide gels at room temperature in 50 mM Tris, pH 8.3, 2 mM EDTA and were detected by autoradiography. For Supershift analysis, 1 μg of the respective antibody (wherever indicated) was included in the DNA protein-binding reaction.

### Real-time PCR

Real time PCR was performed as described previously [25,26]. Briefly, total RNA from cells was isolated with Trizol (Gibco) according to the manufacturer's protocol. Real-time PCR was conducted using a Bio-Rad iCycler (iCycler iQ Multi-Color Real-Time PCR Detection System; Bio-Rad, Hercules, CA). Single stranded cDNA was synthesized from RNA isolated from untreated, LPS/β-amyloid-treated cells in the presence or absence of AICAR using the Superscript preamplification system for first-strand cDNA synthesis (Life Technologies, Gaithersburg, MD). Total RNA (5 μg) was treated with 2 U DNase I (bovine pancreas; Sigma) for 15 min at room temperature in 18 μl volume containing 1× PCR buffer and 2 mM MgCl_2_. It was then inactivated by incubation with 2 μl of 25 mM EDTA at 65°C for 15 min. Random primers were added (2 μl) and annealed to the RNA according to the manufacturer's instructions. cDNA was prepared using poly-dT as a primer and Moloney murine leukemia virus reverse transcriptase (Promega) according to manufacturer's instructions. The primer sets used were designed and synthesized by Integrated DNA technologies (IDT, Coralville, IA). The primer sequences are: for glyceraldehyde-3-phosphate dehydrogenase (GAPDH), forward 5'-CCTACCCCCAATGTATCCGTTGTG-3' and reverse 5'-GGAGGAATGGGAGTTGCTGTTGAA-3'; IL-1β, forward 5'-GAGAGACAAGCAACGACAAAATCC-3' and reverse 5'-TTCCCATCTTCTTCTTTGGGTATTG-3'; TNFα, forward 5'-CTTCTGTCTACTGAACTTCGGGGT-3' and reverse 5'-TGGAACTGATGAGAGGGAGCC-3'; and iNOS, forward 5'-GGAAGAGGAACAACTACTGCTGGT-3' and reverse 5'-GAACTGAGGGTACATGCTGGAGC-3'. IQTM SYBR Green Supermix was purchased from Bio-Rad. Thermal cycling conditions were as follows: activation of iTaq DNA polymerase at 95°C for 10 min, followed by 40 cycles of amplification at 95°C for 30 sec and 55–57.5°C for 30 sec. Levels were expressed as arbitrary units normalized to expression of the target gene relative to GAPDH.

### Cytokine assay

The levels of TNF-α, IL-1β, and IL-6 were measured in culture supernatant by ELISA using protocols supplied by the manufacturer (R & D Systems).

### Transcriptional assays

Primary astrocytes were transiently transfected with NF-κB-, or C/EBP-luciferase reporter gene with β-galactosidase by Lipofectamine-2000 (Invitrogen) according to the manufacturer's instructions. pcDNA3 was used to normalize all groups to equal amounts of DNA. Luciferase activity was determined using a luciferase kit (Promega).

### Cell viability

Cytotoxic effects of various treatments were determined by measuring the metabolic activity of cells with MTT and LDH release assay (Roche).

### Studies on phaeochromocytoma (PC-12) cell neurite extension

Rat phaeochromocytoma (PC-12) cells were plated on 60-mm Petri dishes precoated with 10 mg/ml poly-D-lysine and cultured in Kaighn's modified medium containing 20% Horse serum and 2% FBS, 100 U/ml penicillin, and 100 mg/ml streptomycin (All from GIBCO-BRL) for ~12 h. The cells were then incubated in low-serum media (2% horse serum and 1% bovine calf serum) containing NGF (50 ng/ml) for 48 h before challenging them again with NGF (50 ng/ml) either in the presence or absence of astroglial LPS-conditioned medium and/or AICAR. The cells were then evaluated after 4 days of stimulation by phase contrast microscopy (Olympus). The images obtained were adjusted to set to a color background for clarity using Adobe Photoshop software (version 7). Scoring for neurite outgrowth of PC-12 cells was performed as described previously by Dikic *et al.*[27]. Briefly, neurite lengths greater than 100 μM were taken into consideration and were scored and compared with relevant controls.

### Statistical analysis of the data

All data are expressed as means + SEM. All necessary comparisons were carried out using the Tukey-Kramer multiple comparison test. Statistical differences at p < 0.05 were considered significant. The densitometric data for iNOS and MnSOD, and for all phosphorylation blots are expressed on an arbitrary scale.

## Results

### AICAR attenuates LPS- and Aβ peptide-induced expression of cytokines and iNOS, and NO production in glial cells

**Figure 1:**
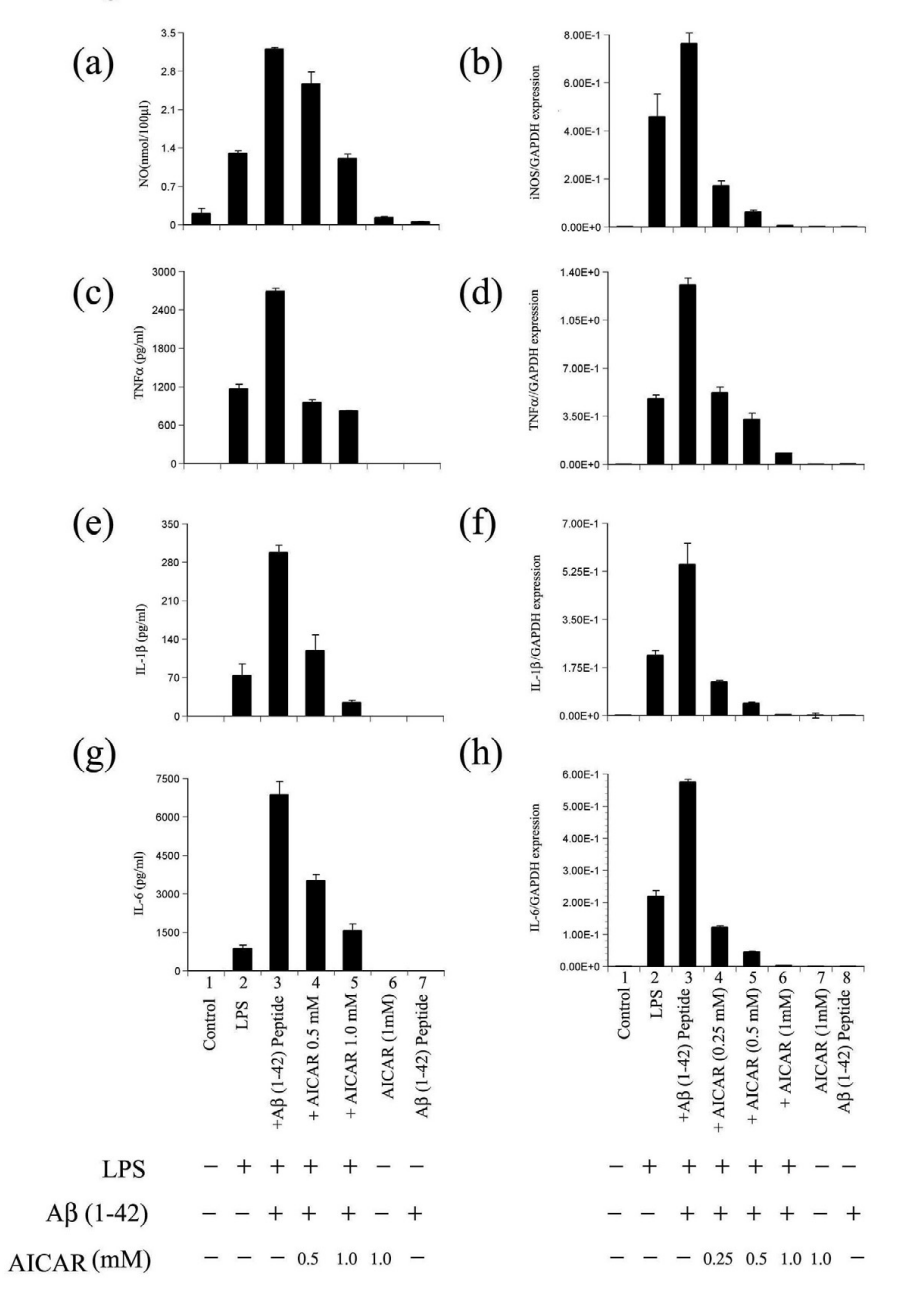
AICAR inhibits LPS- and Aβ peptide-induced cytokine production. Astrocyte-enriched glial cells (mixed glial cells) were pre-incubated with different concentrations of AICAR (as indicated) for 4 h and were stimulated with 1 ng/ml LPS ± Aβ peptide (1–42) (15 μM) as shown. After 18 h of incubation, concentrations of NO, TNF-α, IL-1β, and IL-6 released into the culture medium were measured using ELISA (left panel figs. a, c, e and g). Alternatively the cells were harvested for RNA by extraction with Trizol (see methods) and the levels of mRNA for cytokines were measured (See right panel figs. b, d, f and h) by real time-PCR (RT-PCR). Data are expressed as the mean ± SD of three different experiments. *P < 0.001 was considered significant.

**Figure 2:**
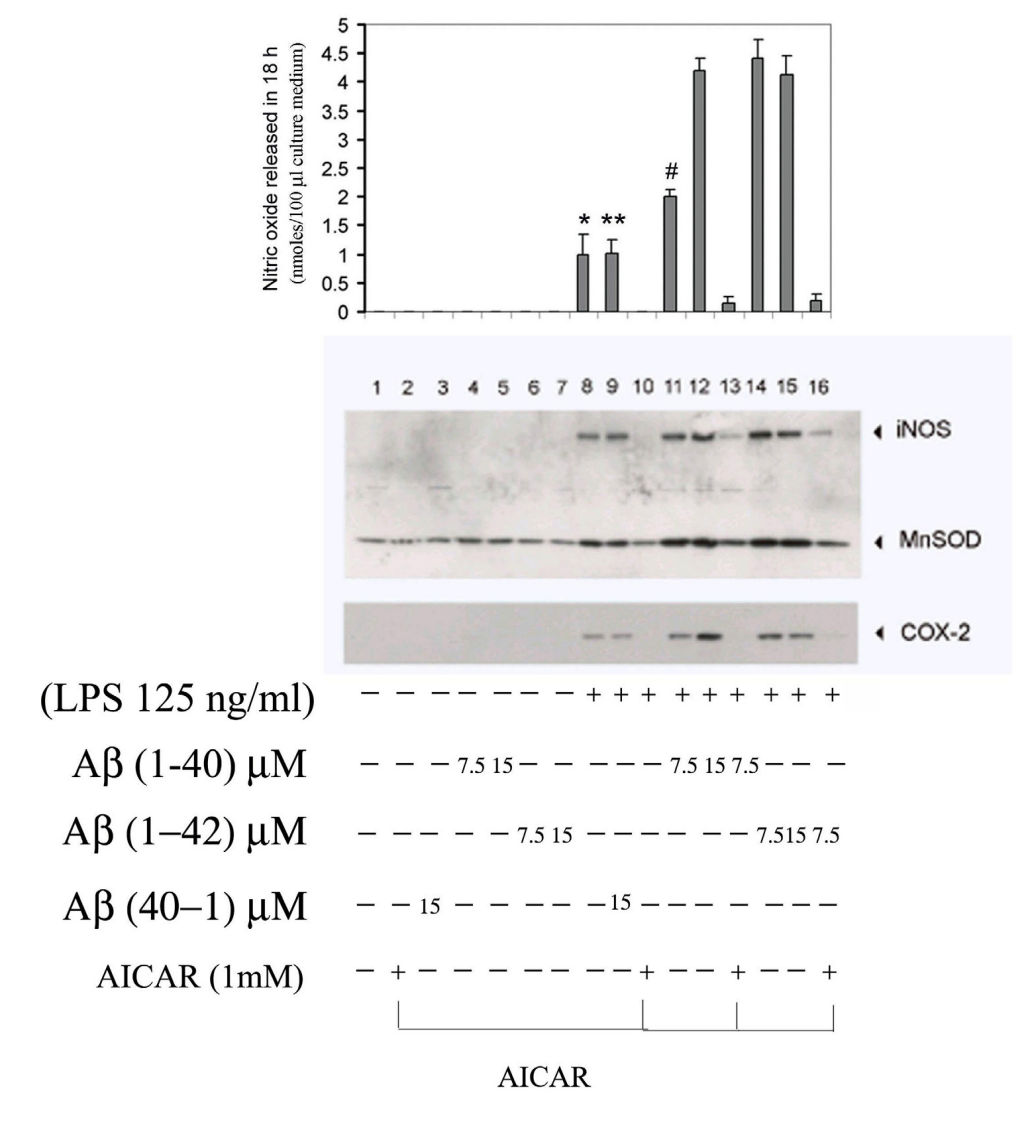
AICAR treatment inhibits LPS + Aβ-stimulated iNOS gene expression and nitric oxide release in glial cells. Cell cultures were pre-incubated with 1 mM AICAR following stimulation by LPS ± Aβ peptides (1–40) or (1–42) in concentrations as indicated. The corresponding reverse peptide (40–1) in lane 3 and 9 served as a positive control in this assay. The production of NO (top) and expression of iNOS, COX-2, and MnSOD was determined in cell lysates, 18 h following treatment, by immunoblot analysis (bottom). Experiments were performed in triplicate and data are means (±SEM). P < 0.05 compared to relative control value was considered significant. However, P value for histograms in lane 8 and 9 (* and **) not significant.

It has been suggested that, in the CNS, activated microglia and astrocytes are linked to neurodegeneration as a result of expression of inflammatory mediators by these glial cells [6,28,29]. Major cytokines implicated in AD (with the major exception of IFN-γ), include TGF-β, TNF-α, IL-1, IL-2, IL-6, IL-10 and IL-12 [3]. In addition to cytokine expression and release, rat primary glial cells are known to express iNOS as well as COX-2. As mentioned earlier, LPS has been routinely used to stimulate/induce the inflammatory cytokine responses in glial cells [7,8,10]. Hence, to mimic the inflammatory responses, rat primary glial cell cultures were treated with LPS ± Aβ (1–42) peptide. As evident from (Fig. 1a,c,e and 1g) and 2, Aβ significantly upregulated the LPS-induced production of cytokines TNF-α, IL-1β, IL-6 and nitric oxide (NO) in glial cells, which is further supported by increases in the expression of mRNA for iNOS, TNF-α, IL-1β and IL-6 (Fig. 1b,d,f and 1h). AICAR attenuated the LPS/Aβ-induced production of TNF-α, IL-1β, IL-6 and NO, and of their mRNA expression, in a dose-dependent manner (Fig. 1a–h).

We have previously reported that SMase-activated ceramide release is redox sensitive and that ceramide-mediated induction of MnSOD and reactive oxygen species (ROS) generation is central to inflammatory responses in glial cells [12,30-32]. Hence expression of MnSOD was routinely evaluated as a ROS-induced stress sensor protein [33]. Figure 2 shows the expression of iNOS, MnSOD and COX-2 in glial cells. Aβ peptide upregulated the LPS-mediated expression of MnSOD. Aβ peptides (1–40) and (1–42) induced the expression of iNOS, COX-2, and MnSOD; but not Aβ (40–1) peptide in reverse sequence. Cells responded to both Aβ peptides (1–40) and (1–42). However, with these two peptides in combination, at equimolar concentrations (7.5 μM each), Aβ (1–42) induced approximately twice the amount of nitric oxide release and correspondingly higher iNOS expression as compared to Aβ (1–40) (lane 11 vs. lane 14). At higher concentrations (15 μM each) of these peptides, the differences in iNOS expression or nitrite production (lane 12 Vs 15) were no longer evident. AICAR treatment blocked the iNOS, COX-2 expression, as well as a nearly normalized expression of MnSOD.

**Figure 3:**
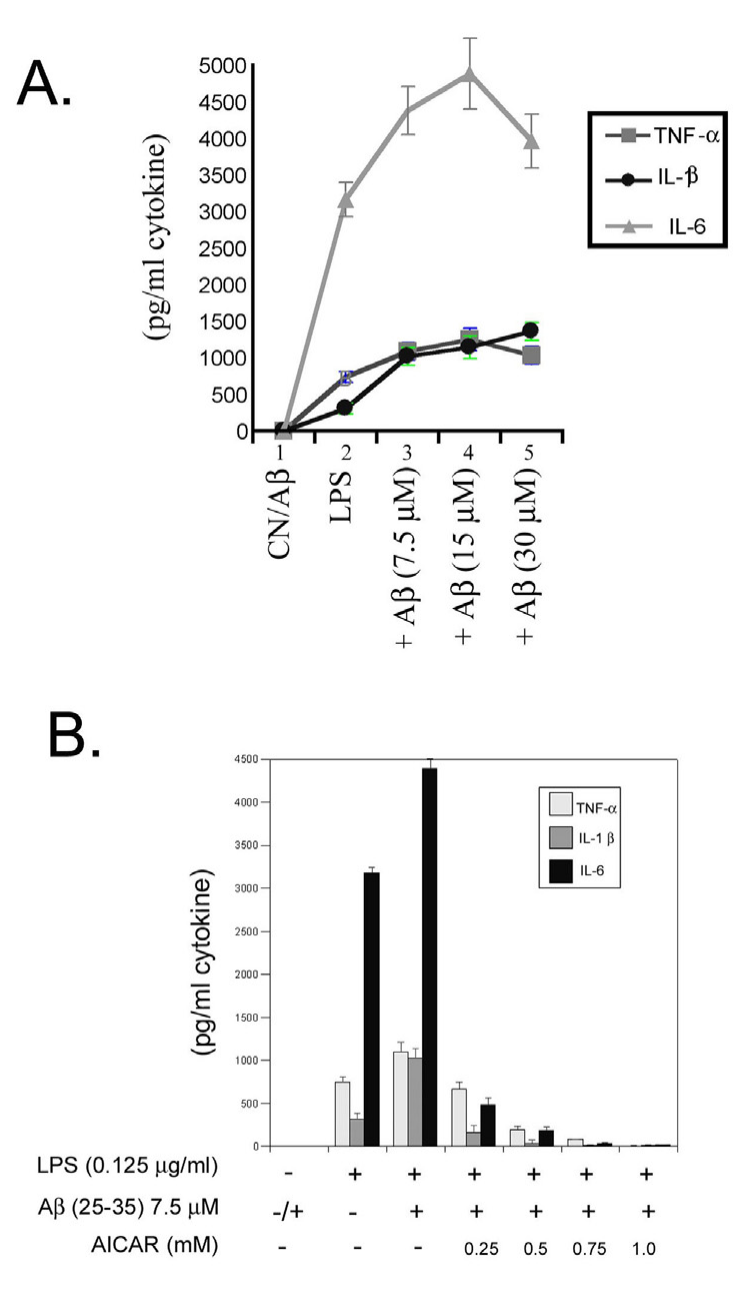
AICAR inhibits LPS- and Aβ (25–35) peptide-induced cytokine production in glial cells stimulated with 125 ng/ml LPS ± 7.5 μM Aβ peptide (25–35). After 18 h of incubation, concentrations of TNF-α, IL-1β, and IL-6 released into the culture medium were measured using ELISA. Fig. 3A. Shows dose-response curves, using LPS and various concentrations of Aβ (25–35) peptide in stimulating cytokine release in glial cells. Fig. 3B shows a dose-response inhibition of cytokine release with various concentrations of AICAR (0.25 to 1 mM) following stimulation with LPS + Aβ (25–35) peptide.

**Figure 4:**
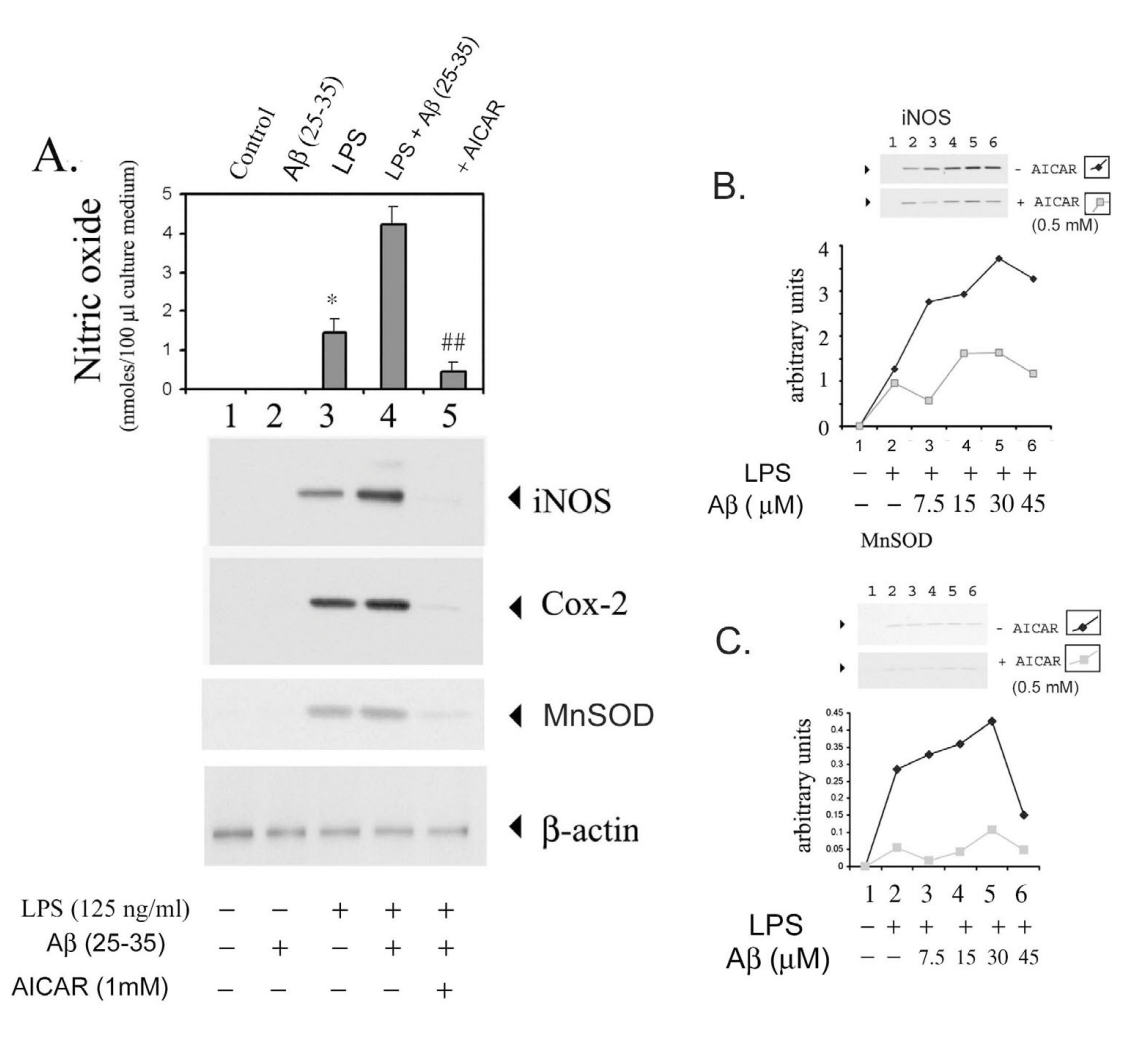
AICAR inhibits LPS- and Aβ- (25–35) induced expression of iNOS, COX-2, and MnSOD in astrocyte-enriched glial cells. Cells were preincubated with 1 mM AICAR for 4 h prior to treatment with either LPS or Aβ (25–35) in concentrations indicated earlier. After 18 h incubation, an aliquot of the medium was used for nitrite measurement as described under Methods. Data are mean ± SD of three different experiments. (A) Cell homogenates were used for western-immunoblot analysis of iNOS, COX-2 and MnSOD. Western immunoblots for iNOS (B) and MnSOD (C) upon treatment of glial cell cultures to LPS and to various concentrations of Aβ (25–35) either in the presence or absence of 0.5 mM AICAR (lane 1 is control, lane 2 LPS alone, and in lanes 3 to 6, Aβ was added to final concentrations of 7.5, 15, 30 or 45 μM, respectively). The protein bands were scanned on a densitometric scanner and represented as a graph (bottom). Experiments were performed in triplicate and data are means (±SEM). *P < 0.05 compared to relative control value was considered significant.

The treatment of glial cells with LPS and Aβ peptide (25–35) elicited a similar inflammatory response in terms of cytokine release and iNOS, COX-2 and MnSOD expression (Fig. 3 and 4) as well as a dose-dependent inhibition with AICAR. Figure 3A shows the dose-dependent expression of TNF-α, IL-1β and IL-6 by Aβ peptide (25–35) and figure 3B shows the dose-dependent inhibition of these cytokines by AICAR. Fig 4 shows inhibition of iNOS and MnSOD expression by 0.5 mM AICAR to a fixed concentration of LPS (125 μg/ml) with various concentrations of Aβ peptide.

Taken together, these studies indicate that Aβ (25–35) peptide induces proinflammatory responses similar to those observed with Aβ (1–40 or 1–42) peptide. Hence, Aβ (25–35) peptide was used in the rest of this study. Cell viability was tested under experimental conditions as described in this study but no toxicity was evident in MTT or in LDH-release assays.

**Figure 5:**
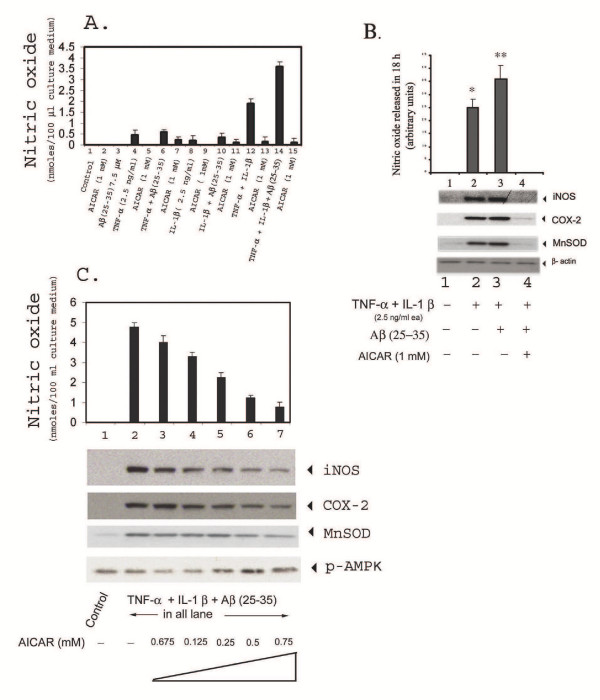
AICAR inhibits TNF-α-, and/or IL-1β- and/or Aβ- (25–35) stimulated iNOS expression and nitric oxide release in astrocytic cellcultures. Cells were pre-incubated with 1 mM AICAR prior to stimulation. Fig. A shows nitric oxide released into the medium upon treatment with cytokines +/- Aβ (25–35) with relevant controls. Figure B shows nitric oxide released into the medium and corresponding western-immunoblot for iNOS, COX-2, and MnSOD, after stimulation with cytokines (TNF-α + IL-1β ± Aβ peptide), either in the presence or absence of AICAR in concentrations used in figure A. Figure C shows a dose-dependent inhibition of nitric oxide production and expression of iNOS, COX-2 and MnSOD proteins, on stimulation with cytokines and Aβ and after pre-incubation with increasing amounts of AICAR, as shown. The increase in p-AMPK protein band (Thr-172) indicates activation of AMPK with increasing concentrations of AICAR.

The observed expression of cytokines TNF-α and IL-1β by activated glial cells (Figures 1 and 3), is consistent with expression of these cytokines in brains of experimental animal models of Alzheimer's and in the brain of Alzheimer's disease patients [3]. We have reported previously that Aβ also upregulates TNF-α/IL-1β-induced iNOS expression and nitrite release [12]. Hence, to study autocrine/paracrine effects, astrocytes in culture were treated with TNF-α/IL-1β. As shown in figure 5, TNF-α/IL-1β treatment led to increased iNOS and COX-2 expression, and nitrite production which was further upregulated by the addition of Aβ (25–35) peptide. The induction of these pro-inflammatory mediators was also significantly attenuated by AICAR (Fig. 5). Further, the increased expression of anti-oxidant enzyme MnSOD in response to TNF-α/IL-1β ± Aβ (25–35), was also markedly reduced upon pre-treatment of glial cells with AICAR (Fig. 5C). From these studies we conclude that AICAR attenuates LPS/cytokine- and Aβ peptide-induced inflammatory cytokine release; and iNOS, COX-2 and MnSOD expression.

### Anti-oxidant functions of AICAR on LPS, Aβ-induced oxidative stress responses

**Figure 6:**
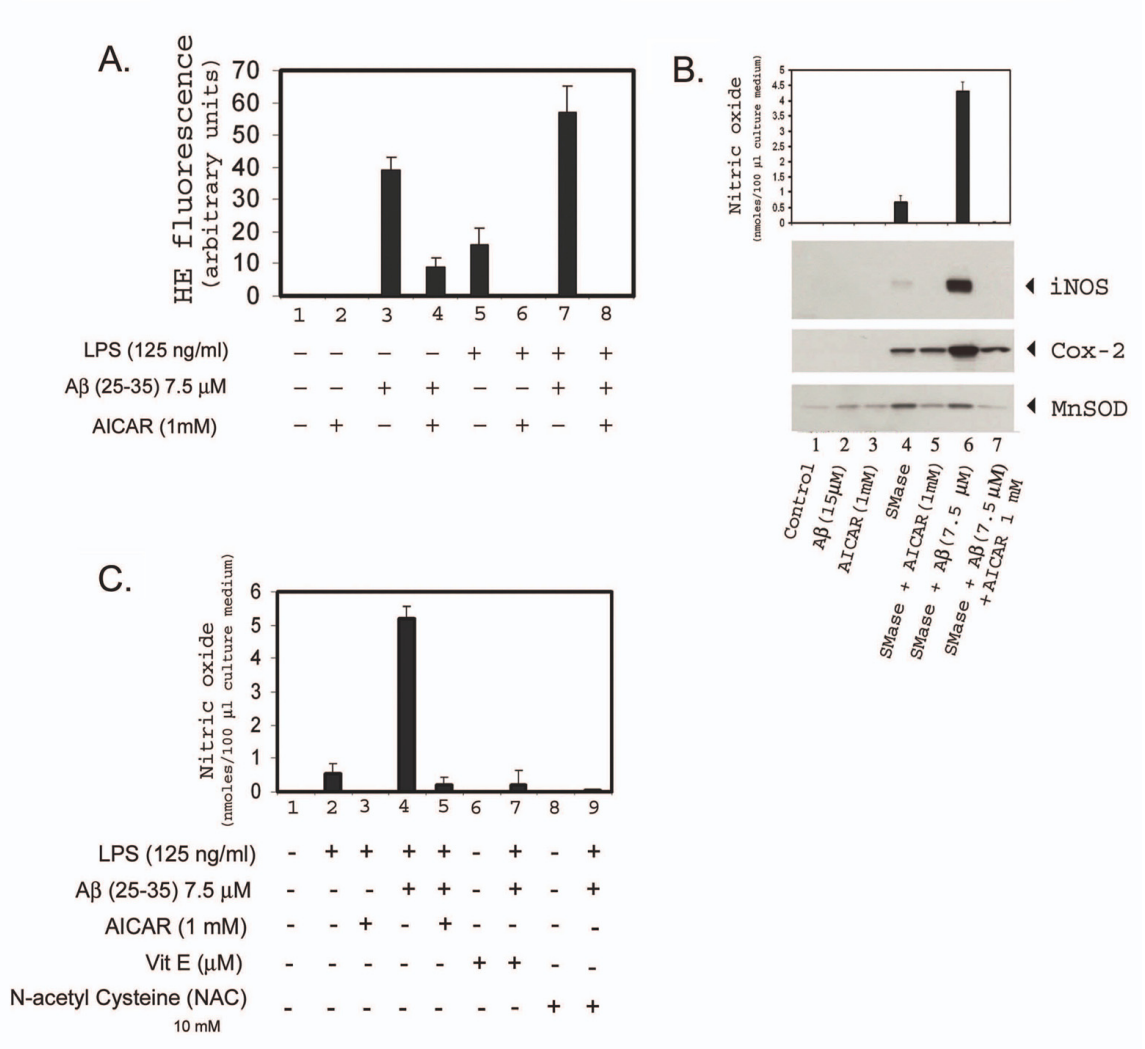
AICAR down-regulates Aβ ± LPS- or sphingomyelinase-induced generation of reactive oxygen species (ROS) and expression of iNOS, COX-2 and MnSOD. Figure A shows LPS ± Aβ- (25–35) induced ROS generation in glial cell cultures. Pre-treatment of glial cells with AICAR inhibits LPS- and Aβ-mediated ROS generation. Cells were pre-incubated in the culture medium with 1 mM AICAR for 4 h prior to treatment with LPS (0.125 μg/ml) and/or 7.5 μM Aβ (25–35). ROS generation was measured by incubating the cells with the fluorescent dye Hydro Ethidene (HE) as described under Methods. Columns where AICAR was added have been shown diagramatically for brevity. Figure B: anti-oxidants (NAC and vitamin E) mediated inhibition of LPS, Aβ-stimulated nitric oxide release in glial cultures. Cells were pretreated with either AICAR (1 mM), vitamin E (20 μM) or N-acetyl cysteine (NAC) (10 mM) 4 h prior to stimulation with 125 ng/ml LPS and Aβ 7.5 μM (25–35). Nitric oxide released into the medium was measured as described under methods. Figure C shows AICAR mediated inhibition of both SMase- and Aβ-stimulated nitric oxide release (top) as well as the expression of iNOS, COX-2 and MnSOD in astrocyte enriched glial cultures (bottom). Cultures were pre-incubated with AICAR 4 h prior to stimulation with SMase (5 units/ml) ± 7.5 μM Aβ (25–35). Nitric oxide produced in the culture medium was measured using 'Greis reagent'. The cell lysates were western-immunoblotted for iNOS, COX-2 and MnSOD expression.

**Figure 7:**
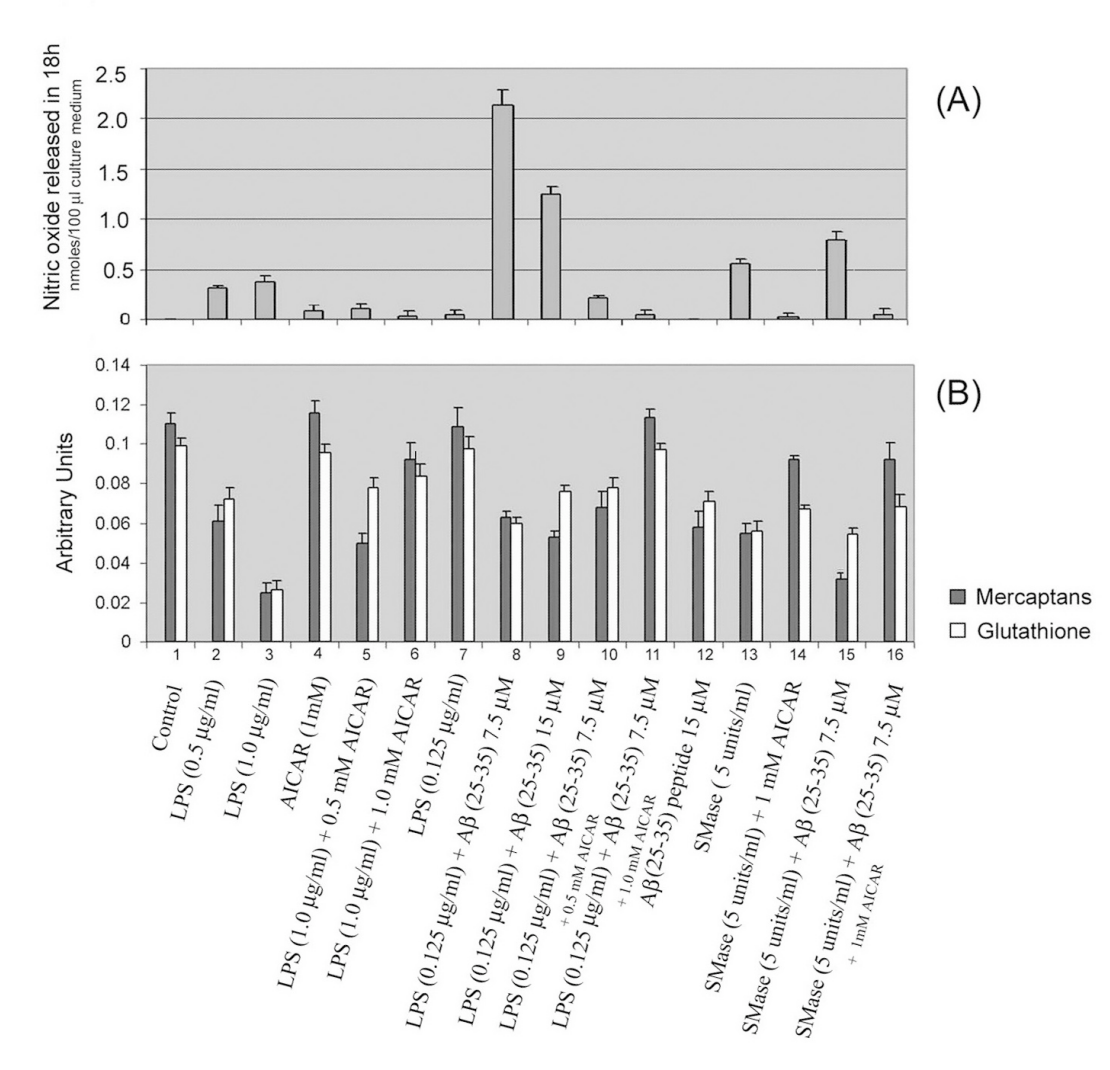
Effect of AICAR in normalization of LPS-, or SMase- and/or Aβ peptide- (25–35) induced decreases in cellular glutathione and total mercaptans (thiol group containing compounds). Figure A shows a histogram plot of NO released after treatment with LPS or SMase and/or Aβ peptide, either in the presence or absence of AICAR (1 mM). Figure B shows corresponding levels of glutathione (in light colored bars) and total mercaptans (dark colored bars).

Earlier studies from our laboratory [12,30], as well as others [20,34] have reported cytokine- or LPS- and Aβ-induced alterations in cellular redox activate the sphingomyelin(SM)-ceramide (Cer) signal-transduction cascade by conversion of sphingomyelin to ceramide in glial cells in culture. This pro-inflammatory cascade of events could be blocked by anti-oxidants such as NAC and vitamin E as well as by neutral sphingomyelinase inhibitor (3-o-methyl sphingomyelin) [12,19,30]. The elevated expression of MnSOD, Cu/ZnSOD, reactive oxygen species (ROS), and reduction in glutathione, indicate altered redox balance upon LPS, Aβ treatment, which was attenuated by vitamin E treatment [35]. Quantification of production of ROS, after treatment of glial cells with LPS/Aβ peptide, using a fluorescent dye-based assay (HE fluorescence) showed an increase in ROS generation, which was blocked by AICAR pre-treatment (Fig. 6A). This inhibition of ROS generation by AICAR treatment possibly blocks the down-stream targets thereby inhibiting the inflammatory gene expression. The generation of ceramide from sphingomyelin was reported to be redox sensitive [30] and ceramide generated by exogenous sphingomyelinase upregulated the expression of iNOS [30]. We observed that SMase – [the enzyme that degrades sphingomyelin (SM) to ceramide (cer)] and Aβ-treatment of glial cells also leads to increased iNOS expression and NO production which is inhibited by preincubating the cells with AICAR (Fig. 6B). This also confirmed our previous observations of the involvement of SM-ceramide cascade-signaling in expression of iNOS and cytokines [12]. These observed alterations of SM-Cer- and ROS-mediated signaling, with LPS/Aβ-induced expression of proinflammatory mediators, by antioxidant activity of AICAR are consistent with our previous observations that LPS/Aβ-induced expression of iNOS and production of NO are blocked by anti-oxidants (vitamin E or NAC) (Fig. 6C) and thus support the conclusion that AICAR functions in blocking the generation of ROS and in turn the SM-ceramide cascade as a suppressor of pro-oxidant activity [12]. Moreover, intracellular glutathione and mercaptans (which includes total cellular thiol group compounds) levels, which showed a decrease with LPS/SMase and/or Aβ peptide treatment, were restored to significant levels with AICAR treatment (fig. 7), thereby confirming AICAR's potential to balance the cellular redox status.

### AICAR treatment upregulates phosphorylation of AMPK, and possibly down-regulates the Pkb/Akt cascade

**Figure 8:**
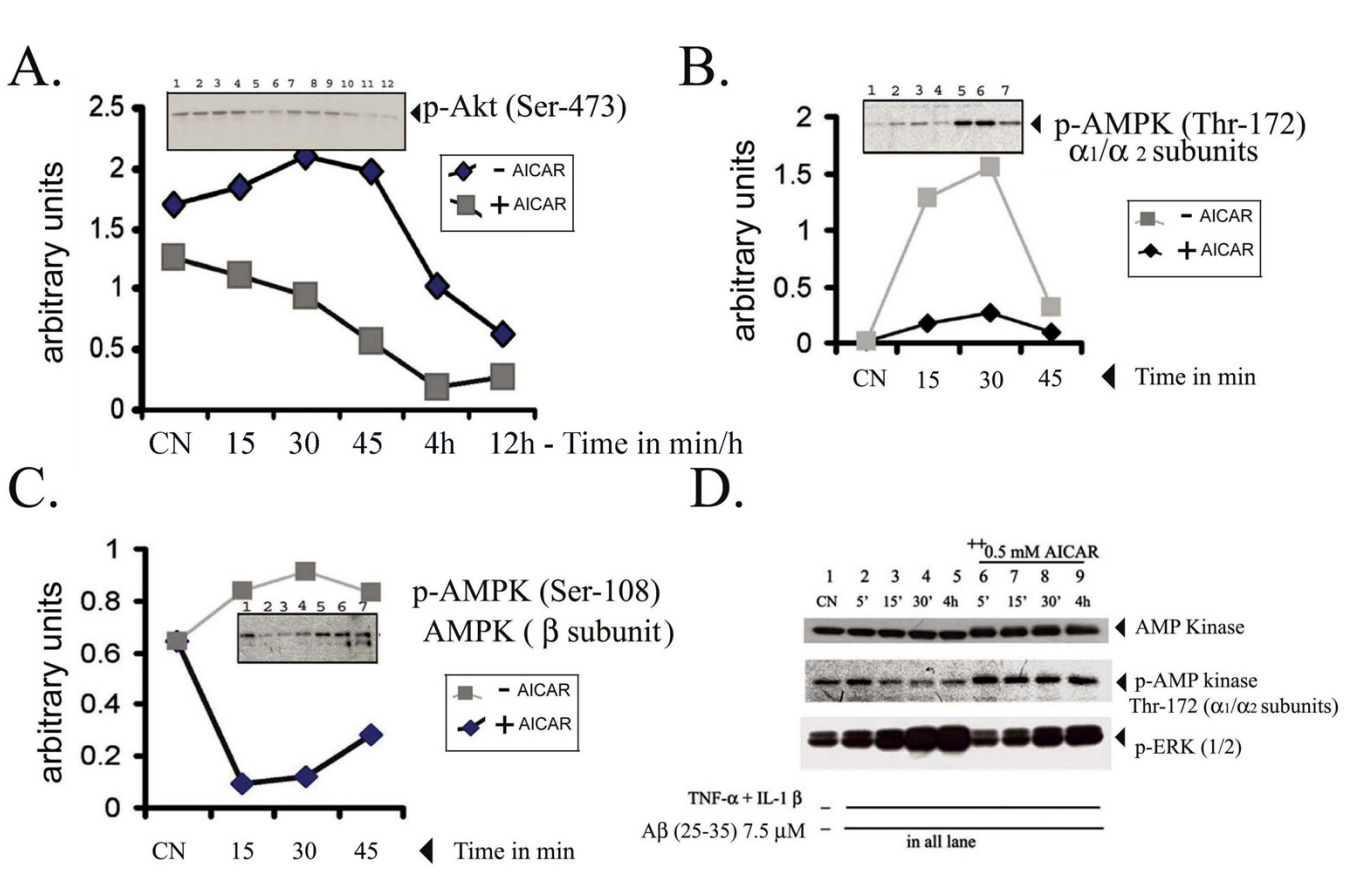
AICAR inhibits LPS-and Aβ-induced activation of Pkb/Akt kinase activity, but activates AMP kinase activity, in astrocyte-enriched glial cell cultures. Cultures pretreated with AICAR or untreated cells were stimulated with LPS (0.125 μg/ml) and 7.5 μM Aβ for the indicated time periods following which cell homogenates were western-immunoblotted for phosphorylated forms of AMPK and Pkb/Akt. Figure 8A shows immunoblots for p-Pkb/Akt proteins. Samples (1 and 7) correspond to control, (2 and 8), (3 and 9), (4 and 10), (5 and 11), (6 and 12) correspond to cells treated with LPS + Aβ +/- 1 mM AICAR for 15 min, 30 min, 45 min, 4 h or 12 h respectively (as shown). Figure B shows phosphor-AMP K (Thr-172 of the α_1_/α_2 _subunits) and Figure C to phosphorylated AMPK (Ser 108) of AMPK β subunit. In Figures B and C samples (1) control (2 and 5), (3 and 6) and (4 and 7) correspond to cells treated with LPS + Aβ peptide (for 15 min, 30 min or 60 min respectively) with or without AICAR pre-treatment. Fig D, shows AICAR-mediated inhibition of TNF-α/IL-1β- and Aβ-induced activation of ERK and activation of AMPK. Cells were pre-incubated with AICAR (1 mM) for 4 h prior to treatment with cytokine and Aβ (25–35). Cell homogenates were prepared at indicated time points and western immunoblotted for either phosphorylated or nonphosphorylated iso forms as shown.

Recent reports from Jhun et al., [36] and a previous study by Morrow et al., [37] reported the involvement of Pkb/Akt kinases via activation of PI-3 kinase in the nitric oxide release pathways in macrophages and in endothelial cells. Hence, we tested the phosphorylation status of Akt upon stimulation with LPS/Aβ, with or without treatment with AICAR. There was an increase in phosphorylation of Ser-473 of p-Akt on stimulation of cells with LPS/Aβ, which was significantly reduced in AICAR-treated cells (Fig. 8A).

We previously reported that AICAR mediates its effects via activation of AMPK and that activated AMPK downregulates pro-inflammatory responses by downregulation of the IKK cascades [19]. Inside the cell (*in vivo*) AICAR is converted to ZMP (an analog of AMP) which activates AMP kinase kinase (AMPKK) which in turn activates AMP kinase (AMPK) by phosphorylation on residues Thr 172 of the α_1_/α_2 _subunits and on Ser 108 of the β subunit of AMPK. AICAR treatment of glial cells activated AMPK as evident from the enhanced intensities of the phospho-specific protein bands of this AMPK at Ser-108 and Thr-172 (Fig. 8B and 8C). Immunoblot analysis of cytokine- (TNF-α/IL-1β) treated cells showed significantly increased ERK phosphorylation (MAP kinase activation) and Aβ treatment further upregulated this MAP kinase activation (Fig. 8D). AICAR treatment down-regulated cytokine/Aβ-induced activation of MAP kinases. These observations indicate that AICAR activation of AMP kinase by phosphorylation of its catalytic subunits (Thr-172 of α_1_/α_2 _subunits) may possibly down-regulate MAP kinase activation and inhibition of proinflammatory gene expression. However, at present it is not clear how the activation of AMP kinase cascade would mediate reduced activation of the MAP kinases.

### AICAR inhibits LPS- or SMase- and Aβ (25–35)-induced NFκB, AP-1, C/EBP and CREB binding activity

**Figure 9:**
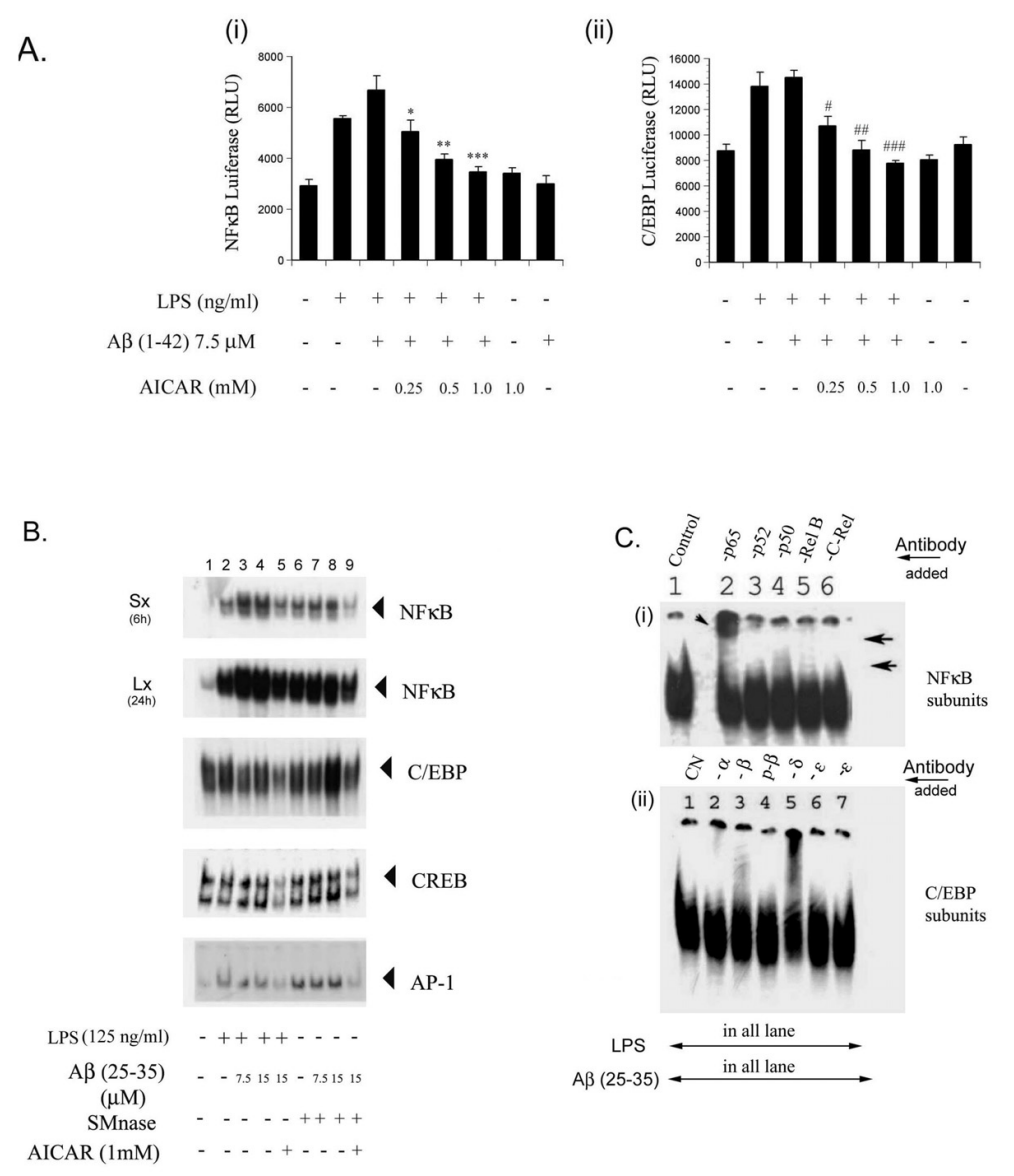
(A) AICAR inhibits LPS- and Aβ-induced activation of NFκB, AP-1 CREB and C/EBP transcription factors. NFkB luciferase and C/EBP luciferase activities in glial cells transfected for NFkB luciferase (i) or C/EBP luciferase following stimulation with LPS and Aβ (1–42) peptide (figure A) were measured as described under legends to figure 1. Experiments were performed in triplicate and data are expressed as mean ± SEM. *P < 0.05 compared to relative control value was considered significant. AICAR inhibited the LPS- or SMase- and Aβ-induced NFκB, C/EBP, CREB and AP-1 binding activity as seen by EMSA (figure B). EMSA was carried out using the nuclear extracts prepared from astrocyte-enriched glial cells after treatment with LPS or SMase and Aβ for 1 h either in the presence or absence of AICAR. In case of EMSA for NFκB, for clarity, the dried gel was exposed for autoradiography either for longer (24 h) or for shorter (6 h) periods. The top shows the picture of the autoradiogram of the shorter exposure time (Sx) and the bottom shows the longer exposure time (Lx). In figure B(i) polyclonal IgGs specific for NFκB subunits -p65, p52, p50, RelB or cRel were used for supershift experiments with nuclear extracts from LPS, Aβ-treated (1 h) glial cells for binding to γ-^32^P-labeled NFκB oligomer. Note the supershifted complexes in lanes 2, 4 and 5 (correspond to -p65, p50 and -RelB proteins). In figure (ii) lanes 2–7, polyclonal IgGs specific for C/EBP α, β, p-β, δ and ε were used in supershift experiments with nuclear extracts from LPS, Aβ-treated (1 h) glial cells for binding to γ-^32^P-labeled C/EBP oligomer. ε antibodies from two different stocks were tested (in lanes 6 and 7). Note the supershifted complexes in lanes 2, 3 and 5 corresponding to -α, β and -δ subunits of C/EBP.

Transcription factors such as NFκB, AP-1, CREB and C/EBP are often the downstream targets of MAP kinase signaling cascades, for the transactivation of genes expressed under proinflammatory conditions [12,38,39]. These transcription factors have consensus sequences in the promoter regions of proinflammatory genes such as iNOS, COX-2, MnSOD as well as those of cytokines [38]. Therefore, we investigated the possible role of these transcription factors in AICAR-mediated regulation of expression of proinflammatory genes. Cell cultures transiently transfected with expression vectors for NFκB-luciferase or C/EBP-luciferase, upon stimulation with LPS/Aβ (1–42) peptide upregulated luciferase activity, a reflection of the activation of these transcription factors. Treatment with AICAR showed dose-dependent attenuation of these luciferase activities (Fig. 9A). To further confirm these observations, we performed EMSA for activation of these transcription factors. Aβ treatment upregulated the LPS- or SMase-induced binding of these transcription factors and this enhanced binding activity was blocked by AICAR treatment (Fig. 8B). NFκB/IKK-mediated transcriptional activity involves different subunits of NFκB (RelA/p65, c-Rel and Rel B or the NFκB p50 and p52 heteromeric combination) [38,40]. Supershift analysis of NFκB using antibodies to various subunits of NFκB demonstrated the possible participation/involvement of p65, p52 and Rel B subunits in the NFκB complexes [Fig. 9(c-i)]. C/EBP β and δ are reported to be the key regulators in the pro-inflammatory cascades in glial cells surrounding the amyloid plaques in Alzheimer's disease brains [12,39]. Similar analysis using antibodies to several different C/EBP subunits revealed the involvement of C/EBP α, β and δ components in LPS/Aβ peptide-induced activation of C/EBP (Fig. 9C-ii).

### AICAR attenuates the inhibition of neurite outgrowth in PC-12 cells by astroglial conditioned medium obtained from glia following stimulation with LPS and Aβ (25–35)

**Figure 10:**
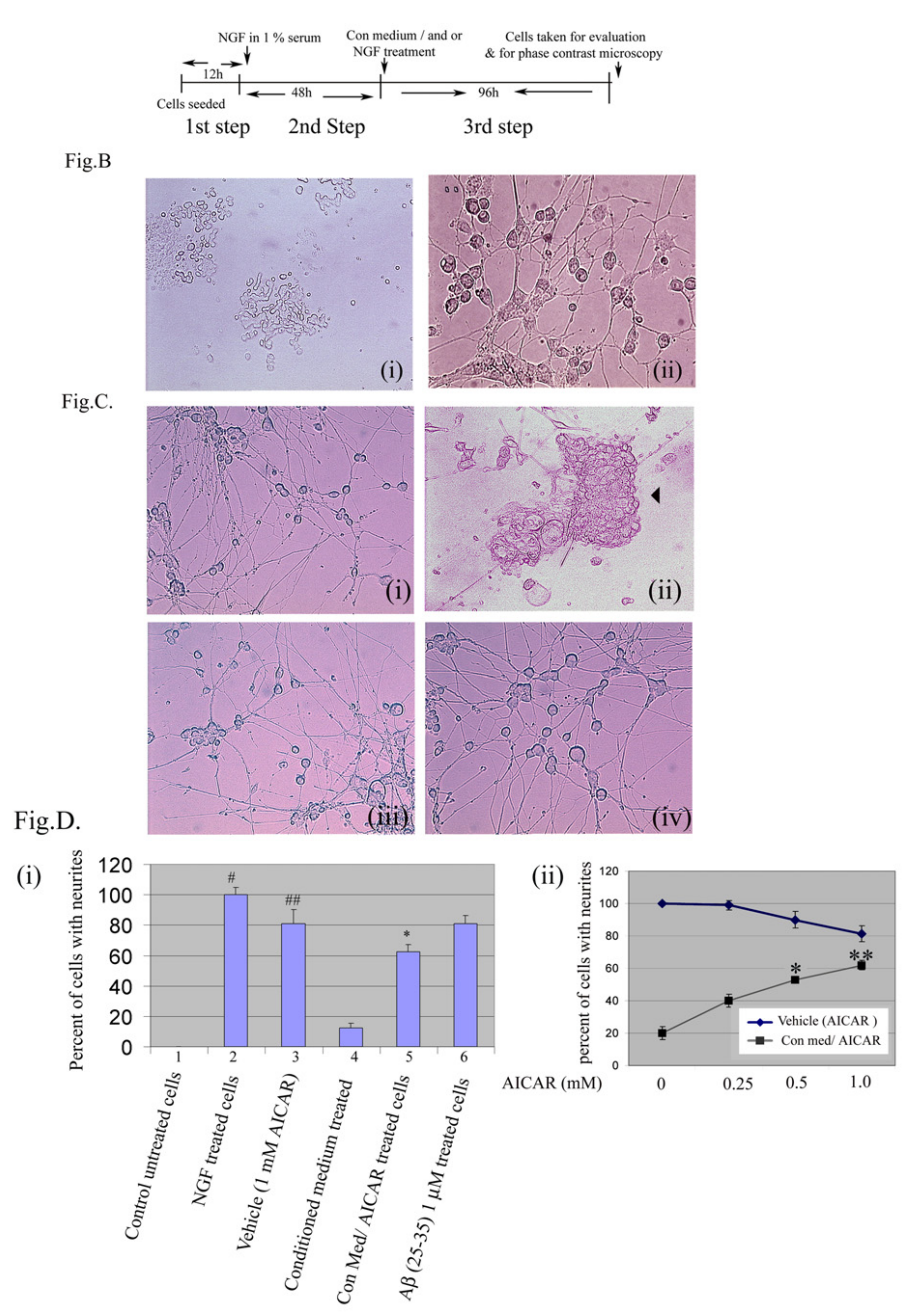
AICAR promotes PC-12 cell neurite extension following stimulation with NGF in the presence of glial conditioned medium. Figure A shows the scheme of treatment using glial cell-conditioned medium (see Methods) either in the presence or absence of AICAR (1 mM). Figures B shows phase contrast micrographs of PC-12 cells of control untreated cells and NGF differentiated PC-12 cells. Where figure B(i) shows control untreated cells (40× magnification) and treated with NGF (50 ng/ml) form neurites reminiscent of axonal network in neurons as shown in figure B (ii) at 40× magnification. AICAR protection against glial conditioned medium is demonstrated in figure 10C where figure (i) is vehicle (control) NGF + 1 mM AICAR treatment. Inhibition of NGF-induced neurite outgrowth observed on treatment with glia-conditioned medium (from LPS, Aβ treatment) showing aggregation of cells (ii) and its reversal in AICAR pre-treated and NGF- and conditioned medium-challenged cells (iii), (all at 20× magnification). Aβ (25–35) (1 μM) treatment was performed as one of the controls (iv) 20× magnification. Experiments were carried out in triplicate. Figure D shows a histogram plot of neurite lengths greater than or equal to 100 μm in the above experiment in vehicle-treated, condition medium-treated, AICAR-treated or Aβ- (25–35) treated PC-12 cells. Data shown are mean ± SEM. # p < 0.5 was considered significant. Figure E shows the effects of different concentrations of AICAR in the presence of fixed amount of conditioned medium on neurite outgrowth. AICAR was tried at three different concentrations against condition medium challenged cells. Values shown are relative percentage values. The neurite outgrowth was recorded after 96 h of NGF stimulation. Quantitative analysis of neurite outgrowth **is **from ~300 cells. The data was plotted as mean ± SD from three experiments. *p < 0.5 was considered significant. P values for Conditioned medium/AICAR treated samples * and ** significant.

Accumulation of amyloid-β protein, a pathological hallmark of AD, also contributes to many alterations of neuronal structure leading to axonal loss and consequent to neuronal cell loss [41,42]. To further investigate the role of AICAR as a neuroprotective agent against cytokines and inflammatory mediators released by glial cells (cytokines, prostanoids and nitric oxide), we studied the effect of conditioned media from LPS/Aβ peptide-treated glial cells on NGF-induced neurite extension of PC-12 cells. The scheme of treatment is described in figure 10A; and under 'Methods'.

PC-12 cells (Fig. 10), following stimulation with NGF, showed extensive neuronal growth forming a network reminiscent of neuronal extensions (Fig 10B). They tend to form extensive neurites that appeared to be connected well to adjacent cells. Challenging the cells with pre-conditioned medium from glial cells led to extensive loss of NGF-induced neurite extension and also to cell clustering and cell death (Fig. 10C). AICAR treated cells (vehicle) without the conditioned media on average showed 20% fewer neurites than control untreated cells (Fig. 10D). However, conditioned medium-challenged cells showed greater than 80% loss in neurites on the average, whereas, AICAR treatment reduced this loss to 25% loss of neurites. To rule out the possibility that Aβ (25–35) peptide from the conditioned medium may be a contributing factor in the observed loss of neurites, Aβ (25–35) (1 μM), treated PC-12 cells showed ~18% loss in neurites as compared to greater than 80% loss in conditioned medium treated cells. AICAR treatment at concentrations of 0.25, 0.5 and 1 mM led to 40-, 53- and 62-percent protection against neurite loss, respectively (Fig. 10E). However, higher concentrations of AICAR (2 mM) were found to be toxic (data not shown). In a similar set of experiments we observed similar protection of neurite extension by AICAR against cytokines (TNF-α and IL-1β)/Aβ (7.5 μM) (data not shown here).

## Discussion

We have previously reported that LPS/Aβ-induced expression of proinflammatory mediators (e.g. iNOS) is mediated via the SM-ceramide-associated cellular redox signaling cascade [12,32]. This study reports activation of AMPK by AICAR, and its possible down-regulation of LPS- or cytokine/Aβ-mediated signaling events associated with cellular oxidative stress and inflammatory activity. These conclusions are based on the following observations. The anti-oxidant functions of AICAR are evident from the observations that AICAR blocks LPS- and LPS ± Aβ-induced ROS generation (Fig. 6) as well as nitric oxide release, very similar to that seen with other anti-oxidants such as NAC or Vitamin E [12,43]. Moreover, MnSOD expression (the mitochondrial oxidative stress sensor), was upregulated along with proinflammatory cytokines, iNOS, and COX-2 in LPS/Aβ-stimulated cells and their expression was down-regulated following AICAR treatment (Figs 2, 3, 4, 5, 6). These antioxidant and anti-inflammatory functions of AICAR (Figs 1, 2, 3), its associated protective effects, and its promotion of neurite outgrowth extension by PC-12 cells (Fig. 10) exposed to glia-conditioned media (and hence to inflammatory mediators secreted by activated glial cells) indicate that AICAR may provide protection against inflammatory mediators (cytokines, NO and O_2_^•-^) and Aβ-mediated toxicity to neurons in AD. In cell culture studies (*in vitro*) AICAR is effective in attenuating the inflammatory process at 0.5–1 mM, and up to 2 mM with no toxicity observed. We have also tested the efficacy of AICAR in attenuation of ischemia-reperfusion injury in a canine model of autologous renal transplantation at 50 mg/kg body weight [44], an animal model of experimental autoimmune encephalomyelitis [45], an animal model of multiple sclerosis at 100–500 mg/kg body weight, and LPS-induced neurotoxicity in rats at 100 mg/kg body weight [19], with no side effects. This points to the *in vivo *beneficial effects of this compound against inflammatory immune response mechanisms.

AICAR, upon internalization into the cells is immediately phosphorylated by adenosine kinase to AICA riboside monophosphate ZMP (a purine nucleotide) which mimics the effect of AMP without altering the cellular ratio of ATP/AMP and thus activates AMP kinase kinase (AMPKK), and in turn AMPK [16,46]. AMPK is known to regulate glucose transport [47] and its metabolism [48], lower blood pressure, boost liver insulin action [49], ameliorate insulin resistance induced by free fatty acids [18], and regulate protein synthesis [50] and forkhead transcription factor FKHR (FOXO1a) [51]. It is also reported to down-regulate the synthesis of fatty acids as well as cholesterol [52]. These observations indicate participation of AMPK in the regulation of cellular energy metabolism. We have earlier reported on the anti-inflammatory role of AICAR via activation of AMPK in quenching LPS-induced pro-inflammatory responses by blockade of MAP kinase and IKK α/β-signaling cascades [19]. AICAR treatment activated AMP kinase activity, and antisense oligonucleotides for AMPKKα as well as expression of dominant negative cDNA of AMPKα in glia reversed the AICAR-mediated inhibition of iNOS gene expression in response to LPS treatment [19]. These above studies demonstrate AICAR attenuation of LPS- or cytokine/Aβ-induced expression of inflammatory mediators (e.g. iNOS, COX-2 and cytokines) by inhibiting the activation of transcription factors (NFκB and C/EBP) required for induction of the inflammatory process. Moreover, the current study highlights yet another novel function of AICAR in protection of neurite outgrowth against the toxicity of inflammatory mediators secreted by activated microglia and astrocytes. This protection of neurite growth may in part be mediated via the energy-(ATP) saving mechanisms of AMPK since activation of AMPK is known to shut or slow energy consuming reactions in the cell [16].

**Figure 11:**
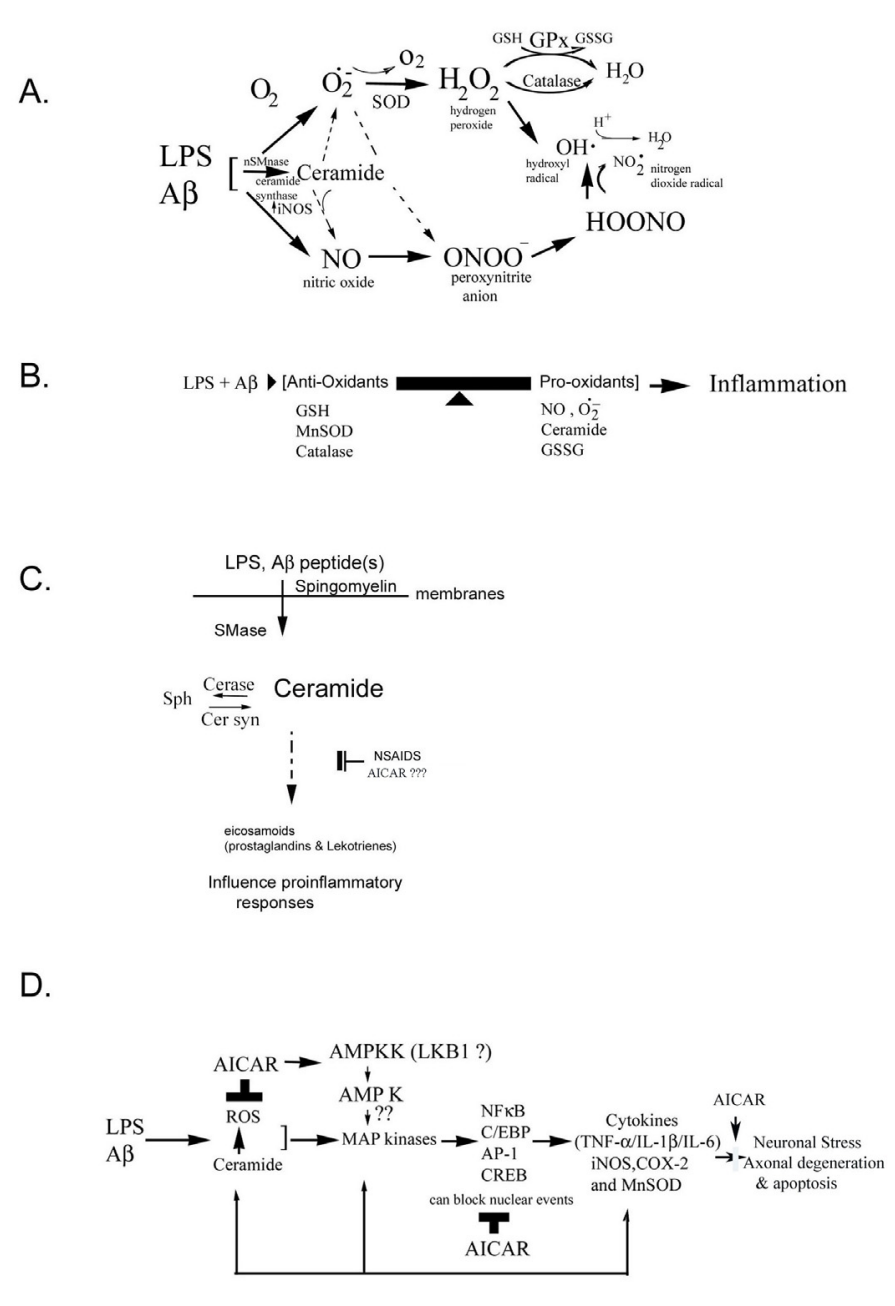
Figure A: Possible anti-oxidant mechanisms involved in the LPS, Aβ-induced ceramide generation leading to superoxide anion formation, nitric oxide release and peroxynitrite generation. Figure B: inflammation is possibly triggered as a result of imbalance in the radical generating systems and radical scavenger systems creating an oxidative stress, thus leading to the formation of nitric oxide and superoxide anion generation, and thereby depleting cellular anti-oxidants. Figure C. Putative role of ceramide in eicosanoid synthesis. Ceramide generated as a result of LPS- and Aβ-peptide induced SMase activation, leads to the release of eicosanoids. Eicosanoids (leukotrienes and prostaglandins) thus generated may perhaps potentially enhance or ameliorate the cytokine-induced pro-inflammatory responses or *vice versa*. Non steroidal anti-inflammatory drugs (NSAIDs) as well as COX inhibitors block these responses. Figure D. Overall scheme in the LPS, Aβ-induced pro-inflammatory signaling cascade involving cytokine release thereby leading to the expression of iNOS, COX-2 and MnSOD. The anti-inflammatory effect of AICAR is perhaps a result of its multiple regulatory roles. However, AICAR blockade of ROS generation keeps the redox balance in check, thereby inhibiting the inflammatory signaling cascade.

Alterations in cellular redox appear to be central to inflammatory events associated with amyloid toxicity (Fig. 11) [12,20,43]. Cytokines as well as Aβ peptides are known to perturb the intracellular redox state via generation of reactive nitrogen species (RNS; NO, ONOO^-^) and reactive oxygen species (ROS; O2^-^, OH^- ^and H_2_O_2_) [53,54] and, more importantly, by reducing cellular thiols [glutathione and other mercaptans (total thiol group containing compounds)] [35]. Glial cells treated with LPS and Aβ showed significantly reduced intracellular levels of glutathione and mercaptans. However, AICAR treatment restored intracellular thiols (Fig. 7). Similarly, MnSOD expression was nearly normalized in AICAR-treated cells (Figs 2, 3, 4, 5, 6). These findings support the idea of antioxidant/ anti-inflammatory functions of AICAR and thereby the potential of AICAR as possible therapy for inflammatory disease processes.

Details of signal transduction pathways that mediate the neurotoxic effects of β-amyloid on neurons and on glia remain elusive. However, glial biology in relation to neuro-inflammatory responses is important for the following reasons: a) Glial cells out number neurons; b) Glia are involved in the upregulation of cytokines and iNOS and thus may participate in chronic β-amyloid-induced activation of astrocytes observed in AD [55]. Astroglia-released cytokines can further activate surrounding astrocytes which may be necessary to phagocytose excessively generated amyloid. The possible role of NO in neuronal damage is supported by the protection observed with NOS inhibitor (N^g^-nitro-L-arginine methyl ester [L-NAME]) in Aβ- (1–42) induced selective loss of cholinergic neurons [13,29,56]. Furthermore, induction of iNOS following direct injection of β-amyloid into rat brain also supports a role for NO-induced toxicity in Aβ-mediated neurotoxicity [55]. Release of inflammatory cytokines, iNOS, ROS and NO may cause direct stress to neurons. However ROS and NO generation in the same environment can have potentially detrimental effects, due to the formation of peroxynitrite radicals which have the potential to cause neuronal stress and apoptosis (Fig. 11A and [57]). In rodent model studies, astrocytes are reported to damage neurons through NO production [3]. Hence our findings described in this study, documenting inhibition of production of both NO and ROS by AICAR, suggest AICAR/AMPK-mediated protection against cytokine/Aβ-induced oxidative stress/neurotoxicity in AD.

Several studies have indicated that use of non-steroidal anti-inflammatory drugs (NSAIDs) may delay the onset and/or slow the cognitive decline in AD [58,59]. COX-2 is an important enzyme in the PLA2 pathways for the synthesis of various eicosanoids (Fig. 11 and [55]). There is evidence that COX-2 may exacerbate neuronal injury in a variety of diseases [58]. It has been reported that ceramide generated by activated SMase activates cPLA2 cascades leading to enhanced COX-2 expression and hence to the release of eicosanoids [55,60]. ROS are yet another by-product of the conversion of arachidonic acid to prostanoids (prostaglandins and leukotrienes), and perhaps one of the leading contributors of neuronal cell death [61]. COX-2 over-expression has been reported in apoptotic neuronal cell death, and inhibition of COX-2 activity has been reported to protect neurons against excitoxicity in ischemia- and seizure-induced injury [58,62]. Specific COX-2 inhibitors have also been reported to suppress COX-2 activity and to reduce neuronal cell death in the CNS of animal models of cerebral ischemia [63,64]. Upregulation of COX-2 expression in an Alzheimer's mouse model [65] and in cell culture studies has been reported in response to Aβ toxicity [66], indicating the potential of selective COX-2 inhibitors as neuroprotective agents in AD [58,59]. Since, iNOS and COX-2 are important components of the post-lesion inflammatory cascade in various types of brain damage [67], the observed suppression of Aβ and LPS/cytokine-induced COX-2/iNOS expression in glial cell cultures indicates the potential of AICAR to protect against Aβ-induced inflammatory disease process.

## Conclusion

The major themes of ROS and RNS formation associated with the neuroinflammatory processes, and the suppression of these stress mechanisms by antioxidants, continue to yield promising leads for new therapies. Anti-oxidants have been reported to have beneficial effects against Alzheimer's disease [6,20]. Numerous studies in various experimental paradigms of neuronal cell death both *in vitro *and *in vivo*, have shown protection by free radical scavengers including vitamin E, estrogen, ebselen, flavanoids, N-acetyl cysteine, glutathione, α-lipoic acid, etc [20]. The fact that Aβ peptide-associated oxidative damage leads to neuroinflammation, which is effectively attenuated/blocked by AICAR treatment, provides strong evidence that altered redox equilibrium processes are directly related to neuroinflammation.

Disease progression in Alzheimer's disease (AD) often causes massive neuronal stress, contributing to the loss of cognitive function observed in the disease. Many brain regions in patients with AD show changes in axonal and dendritic fields, dystrophic neurites, synapse loss, as well as neuronal loss [41]. Accumulation of amyloid-β protein and tau-induced changes (in the form of 'neurofibrillary tangles') are pathological hallmarks of the disease and are believed to contribute to many of these alterations of neuronal structures [42]. More so, areas of the brain displaying high degrees of plasticity are particularly vulnerable to degeneration in Alzheimer's disease. Perhaps this reflects a loss in the regenerative capacity of the brain, relative to renewed axonal growth, or perhaps a reduced capability of pluripotent stem cells to replace dystrophic neurites. Hence AICAR's potential to aid neurite outgrowth in PC-12 cells challenged with toxic mediators suggests that it may prove beneficial in AD, perhaps leading to functional recovery in these patients. In conclusion, the observed anti-inflammatory and anti-oxidant and neuroprotective functions of AICAR point to the multiple regulatory and therapeutic potentials of this drug for AD.

## List of abbreviations used

AICAR (5-aminoimidazole-4-carboxamide-1-beta-4-ribofuranoside); Aβ (beta amyloid peptide); ROS (Reactive oxygen species); RNS (Reactive nitrogen species); NGF (Nerve growth factor); MnSOD (manganese superoxide dismutase); SDS-PAGE (sodium dodecyl sulfate-polyacrylamide gel electrophoresis); MTT (methylthiazoletetrazolium); EMSA (Electrophoretic mobility shift assay); COX-2 (Cycloxygenase-2); TNF-α (tumor necrosis factor alpha); SMase (Sphingomyelinase); ROS (reactive oxygen species); NFκB (Nuclear factor kappa B); C/EBP (CCAAT enhancer binding protein).

## Competing interests

The author(s) declare that they have no competing interests.

## Authors' contributions

KRA carried out the various experiments, participated in the design of the study and helped draft the manuscript.; AKS and IS participated in the design of the study and helped to draft the manuscript. All authors read and approved the final manuscript.
